# Health promotion for young Brazilian and Portuguese university students in terms of self-perception and self-image: Instagram profile

**DOI:** 10.17533/udea.iee.v43n3e07

**Published:** 2025-10-25

**Authors:** Lia Araruna De Lima, Henriqueta Ilda Verganista Martins Fernandes, Paulo César De Almeida, José Eurico De Vasconcelos Filho, Luísa Maria Da Costa Andrade, Karla Maria Carneiro Rolim, Ana Clécia Jácome Unias, Fernanda Jorge Magalhães

**Affiliations:** 1 Nurse, Master. Email: liaararuna@gmail.com. https://orcid.org/0000-0003-3324-766X liaararuna@gmail.com; 2 Nurse, Ph.D. Porto, Portugal. Email: ildaverganistafernandes@gmail.com. https://orcid.org/0000-0002-8440-3936 Porto Portugal ildaverganistafernandes@gmail.com; 3 Biostatistician, Ph.D. Fortaleza-CE-Brazil. Email: pc2015almeida@gmail.com. https://orcid.org/0000-0002-2867-802X Fortaleza CE Brazil pc2015almeida@gmail.com; 4 Computer Scientist, Ph.D. Fortaleza-CE-Brazil. Email: euricovasconcelos@gmail.com |://orcid.org/0000-0002-6881-0814 Fortaleza CE Brazil euricovasconcelos@gmail.com; 5 Nurse, Ph.D. Porto, Portugal. Email: luisaandrade@esenf.pt https://orcid.org/0000-0002-5715-855X Porto Portugal luisaandrade@esenf.pt; 6 Nurse, Ph.D. Fortaleza- CE- Brazil. Email: karlarolim@unifor.br. https://orcid.org/0000-0002-7914-6939 Fortaleza CE Brazil karlarolim@unifor.br; 7 Nursing student. Fortaleza-CE-Brazil. Email: clecia.unias@aluno.uece.br. https://orcid.org/0009-0004-6796-3319 Fortaleza CE Brazil clecia.unias@aluno.uece.br; 8 Nurse. Ph.D. Email: fernandaj.magalhaes@uece.br. Corresponding author. fernandaj.magalhaes@uece.br; 9 University of Fortaleza (UNIFOR), Brazil. University of Fortaleza Brazil; 10 Cintesis, Rise, Coordinating Professor, Portugal. Portugal; 11 University of Ceará (UECE), Brazil. https://orcid.org/0000-0003-0104-1528 Universidade Estadual do Ceará University of Ceará Brazil; 12 Citinova. Fortaleza Science, Technology and Innovation Foundation, Brazil. Fortaleza Science, Technology and Innovation Foundation Brazil; 13 Porto School of Higher Education in Nursing (ESEP), Portugal. Porto School of Higher Education in Nursing (ESEP) Portugal

**Keywords:** health education, young adults, social network, self-perception, self-image, social networking., educación sanitaria, adultos jóvenes, red social, autopercepción, autoimagen, red social., educação em saúde, jovens adultos, rede social, autopercepção, autoimagem, rede social.

## Abstract

**Objective.:**

To build a scientific content profile with validity evidence on the Instagram social network, focusing on health promotion for Brazilian and Portuguese university students regarding self-perception and self-image.

**Methods.:**

A multi-method study based on Design Thinking in four stages: Knowledge Building (1st Review with 26 studies and Documentary Study with 962 forms); Product Definition (2nd Review with 50 studies and Focus Group with 13 participants); Development (Profile Prototyping); and Evaluation and Delivery (with evidence of usefulness, ease, and acceptability assessed by seven users).

**Results.:**

It was evidenced in 42.3% of the studies that young people have difficulties with the use of alcohol and drugs; among technologies for intervention, multimedia campaigns and social networks were highlighted. The majority (53.8%) of the records expressed the young people's desire to change something about their bodies. The focus group understood the need to intervene in the self-perception and self-image of young people through Instagram. The profile @multi.brasilportugal was created with content about the promotion of self-care, links for theoretical depth, and professional referrals. Regarding usefulness, ease, and acceptability, users considered it extremely likely to be useful for achieving the objective, extremely easy to operate the profile, and quite likely in terms of the clarity and understanding of the interaction with the technology.

**Conclusion.:**

Design Thinking encouraged the creation of an Instagram profile with interaction and the possibility of using scientific content for education and health promotion, especially to improve body positivity and self-esteem.

## Resumen

**Objetivo.:**

Construir un perfil de contenido científico con evidencias de validez, en la red social Instagram, sobre la promoción de la salud de jóvenes universitarios brasileños y portugueses en términos de autopercepción y autoimagen.

**Métodos.:**

Estudio multimétodo basado en el Design Thinking en cuatro etapas: Construcción del conocimiento (1ª Revisión con 26 estudios y Estudio Documental con 962 formularios); Definición del Producto (2ª Revisión con 50 estudios y Grupo Focal con 13 participantes); Desarrollo (Prototipado del Perfil) y; Evaluación y Entrega (con evidencias de utilidad, facilidad y aceptabilidad evaluadas por siete usuarios).

**Resultados.:**

Se evidenció en el 42.3% de los estudios que los jóvenes tienen dificultades con el uso de alcohol y drogas; entre las tecnologías para la intervención se resaltaron las campañas multimedia y las redes sociales. En la mayoría (53.8%) de los registros se expresó el deseo de los jóvenes de cambiar algo en sus cuerpos. El grupo focal comprendió la necesidad de intervenir en la autopercepción y autoimagen de los jóvenes por medio de Instagram. Se construyó el perfil @multi.brasilportugal con contenidos acerca de la promoción del autocuidado, enlaces de profundización teórica y derivación profesional. En cuanto a la utilidad, facilidad y aceptabilidad, los usuarios consideraron extremadamente probable que sea útil para alcanzar el objetivo, extremadamente fácil de operar el perfil y bastante probable en la claridad y comprensión de la interacción con la tecnología.

**Conclusión.:**

El Design Thinking incentivó la creación de un perfil en Instagram con interacción y la posibilidad de usar contenido científico para la educación y la promoción de la salud, especialmente, para mejorar la positividad corporal y la autoestima.

## Resumo

**Objetivo.:**

Construir um perfil de conteúdo científico com evidências de validade, na rede social *Instagram*, sobre a promoção da saúde de jovens universitários brasileiros e portugueses em termos de autopercepção e autoimagem.

**Métodos.:**

Estudo multimétodo baseado no Design Thinking em quatro etapas: Construção do conhecimento (1ª Revisão com 26 estudos e Estudo Documental com 962 formulários); Definição do Produto (2ª Revisão com 50 estudos e Grupo Focal com 13 participantes); Desenvolvimento (Prototipagem do Perfil) e; Avaliação e Entrega (com evidências de utilidade, facilidade e aceitabilidade avaliada por sete usuários).

**Resultados.:**

Evidenciou-se em 42,3% dos estudos que os jovens tem dificuldade quanto ao uso de álcool e drogas; das tecnologias para intervenção ressaltou-se as campanhas multimídia e redes sociais. A maioria (53,8%) dos registros expressou o desejo dos jovens em mudar algo em seus corpos. O grupo focal compreendeu a necessidade de intervir na autopercepção e autoimagem dos jovens por meio do Instagram. Foi construído o perfil @*multi.brasilportugal* com conteúdos a cerca da promoção do autocuidado, *links* de aprofundamento teórico e encaminhamento profissional. Quanto à utilidade, facilidade e aceitabilidade, os usuários consideraram extremamente provável que seja útil para atingir o objetivo, extremamente fácil de operar o perfil e bastante provável na clareza e compreensão da interação com a tecnologia.

**Conclusão.:**

O Design Thinking incentivou a criação de um perfil no *Instagram* com interação e possibilidade de usar conteúdo científico para a educação e a promoção da saúde, especialmente, para melhorar a positividade corporal e a autoestima.

## Introduction

It is estimated that there are currently approximately 1.8 billion young people, which corresponds to almost 25% of the world's population.^1^ For the World Health Organization (WHO), youth is considered to be people between the ages of 15 and 24, which can be broken down into: young adolescents (15 to 19) and young adults (20 to 24).^2^ Promoting the health of this public is considered a challenge, given the important changes they face in their physical, cognitive, emotional and psychosocial context, especially when they enter the university environment, where they undergo personal and social transformations (leaving their parents' home, entering new friendship cycles).^3^ They bring experiences that seek to explore discoveries, sensations, new knowledge and even guarantee participation in social groups. Given this context, it is essential to invest in health promotion strategies using health education and technologies that bring motivation, involve young people in their study, work, family and community environments and bring about changes in behavior.^4^

According to data from the Digital 2024 report, around 79% of young people between the ages of 15 and 24 are connected to the internet globally. Of these, approximately 90% use social networks regularly. This high rate of use reflects the strong presence of this age group and their tendency to use social platforms as a primary means of online communication and interaction. These figures are even higher in developed regions where internet penetration is more pronounced. The most used are *Instagram* (preferred by teenagers and young adults), *YouTube* (most accessed by younger adults) and *TikTok.*^5^The study is justified and relevant in terms of the use of an intervention on a digital social network, since this is the main channel of communication and information-seeking for this age group, with the aim of contributing to the promotion of the health of young university students, directly impacting on their knowledge of self- image recognition and self-perception strategies in order to favor their quality of life as young people. And indirectly, in the medium or long term, on their quality of adult life, by encouraging changes in social and health behaviors that represent risk and/or vulnerability.

In this way, the study aimed to build a profile of scientific content, with evidence of usefulness and ease, on the social network *Instagram*, about the health promotion of young Brazilian and Portuguese university students for self-perception and self-image.

## Methods

Multi-method study with a qualitative approach consisting of four stages, based on the Design Thinking (DT) framework: Stage 1: Knowledge building (1st Integrative Review and Documentary Study); Stage 2: Exploration of gaps (2nd Integrative Review and Focus Group) 3rd stage: Construction and Development (Social Network Prototyping); and 4th stage: Evaluation (validation of evidence of validity and delivery). Design Thinking is defined as a process that aims to create innovative solutions to common problems and has two basic principles: first, it is person-centered, that is, understanding their needs and desires is fundamental. Second, it is necessary to develop and explore a creative *mindset*, asking questions, visualizing possible ideas, creating prototypes, conducting multiple tests.^6^In addition, the stages of *DT* - empathy, problem description, ideation, prototyping and testing - can also be used in nursing practice to promote multidisciplinary teamwork and stimulate the innovation in addressing challenging medical issues.^7^ Held from 2021 to 2022, in a private Higher Education Institution (HEI) in the city of Fortaleza-CE-Brazil and in a Nursing School in the city of Porto, Portugal.

For the first stage, called "Building knowledge", an integrative review (IR) was carried out to identify, in scientific evidence, the technologies that are being used to promote the health of young people/adults, as well as to learn about and analyze their social and health behaviors. The search was conducted in the following databases: *Cumulative Index to Nursing and Allied Health Literature* (CINAHL), *Medical Literature Analysis and Retrievel System Online* (MEDLINE), *Cochrane Library* and Latin American and Caribbean Health Sciences Literature (LILACS). The strategy used combinations of controlled descriptors in Health (DeCS) and the Boolean operator “AND”: Young Adult, Social Behavior, Health-Related Behaviors and Health Promotion, with a final sample of 26 studies. 

To complement the results of the first RI, a documentary study was conducted with 962 forms completed by young university students, 123 of whom were Brazilian and 839 Portuguese, from a multicenter study previously conducted at a Brazilian university and two other Portuguese universities.^8^ The aim was to understand the main changes in the social and health behaviors of these young people. These were young people aged between 16 and 24, who anonymously and voluntarily answered a questionnaire about social and health behaviors, such as: socioeconomic variables, teaching and learning, family relationships, feelings and emotions, sleep and rest, and social determinants of health - alcohol and drug use, food, safety and leisure.

After the completion of the first stage of the study, the second stage was carried out, called Product Definition (with the 2nd IR and a Brainstorming with a focus group), the objective was to explore the gaps among university students in relation to health promotion for self-care. 

To this end, in April 2022, the 2nd IR was prepared to identify the content to be included and discussed in light of scientific evidence on health promotion for young people in terms of their self-perception and self-image (gaps identified as a higher priority). Searches were conducted in the following databases: *Cumulative Index to Nursing and Allied Health Literature* (CINAHL), *Medical Literature Analysis and Retrieval System Online* (MEDLINE), *Ebook Nursing, Web of Science and Scopus*, and the combinations between descriptors and the Boolean operators "*AND*" and "*OR*". Os DeCS foram: Young Adult, Health Promotion, Self-Image, Weight Perception and Health Education. 

At this point, to encourage brainstorming, a qualitative study was added using the Focus Group (FG) technique. The meeting was held remotely via Google Meet®, with the objective of ideation and initial construction of the technological prototype based on DT. The inclusion criteria were: being a university student, having experience in teaching health courses, and having practical experience in assisting young people and/or adolescents. Therefore, the final sample included

13 participants were invited to participate intentionally, for convenience, using the snowball technique. Therefore, two young Brazilian university students and three Portuguese university students; two Brazilian university professors and two Portuguese university professors; and two Brazilian nurses and two Portuguese nurses working in the field of young adult health. It should be noted that two participants were excluded, one Brazilian student and one Portuguese university professor, because they were not present until the end of the meeting, as recommended in the literature.^9^

The FG strategy was used in two moments, using an audio-visual recorder to collect the speeches and then transcribe them. The first meeting investigated the real needs of young university students in terms of health promotion. In the second meeting, the researcher presented a compilation of the requirements/needs to the participants and tried to come up with a suggestion for approaching the target audience with regard to the technology to be built and validated. As part of the planning for this fourth phase, a persona was created (Ana, 19, young university student). Based on this presentation, the nurses, the main researcher and the master's student, together with their supervisor, a doctor, led the FG through the variables related to the self-perception and self-image of young people/adults, in order to continue the process of ideation and prototyping for the construction of the technology. After the discussions involved in the FG and based on the analysis of the participants' speeches, coded by P1, P2, ..., P13, the main requirements for the technology development process were obtained. 

For the third stage of the study, called Development (Profile Prototyping), support was requested from a technology and innovation laboratory at a private in the city of Fortaleza-CE-Brazil. With the team of *designers* and developers, it was possible to build an *avatar* inspired by the persona validated in the ideation and prototyping stage. To build the *avatar*, the researchers created a base model using the *ReadyPlayer®* platform, which was later refined and improved by the team at the lab using the *Zbrush, Blender* and *Mixamo* tools. At the same time as building Ana's *avatar*, a new profile was created on the *Instagram®* social network, as well as educational content in the Canva® program. The aim was to create a page for empowerment, Health Literacy and communication between young people with a dynamic, clear, comprehensive and interactive approach that would meet the needs, especially those related to self-perception and self-image, of young university students.

The fourth and final stage of the study was the evaluation of the *Instagram®* profile, with an analysis of usefulness, ease of use and user acceptance, which were validated in terms of content and tested for reliability and construct validity in two studies involving a total of 152 users and four application programs.^10^ This instrument aims to understand the user's perception of usefulness (capable of being used advantage), ease of use (effortless) and acceptance of the technology for health promotion among young university students.

The technology was applied to five users (two young university students, one Brazilian and one Portuguese, and a Brazilian university professor) to evaluate the health-promoting technology. To this end, they were recruited by verbal invitation, intentionally, for convenience based on initial contact via messaging apps and/or e-mail. The evaluation period was October 2022. These participants were instructed to access *Instagram®*, either through their personal profile or through the professional profile of the private HEI's Master's program, to evaluate the profile built in the previous stage, called *@multi.brasilportugal*.

All ethical aspects were respected, and the study was approved by the Ethics and Research Committee under opinion no. 3.215.533.

## Results

To present the results, we tried to follow the stages of the *DT*, called: "1st Stage of the *DT - Building* Knowledge (IR and Document Study)"; 2nd Stage of the *DT -* Product Definition (*Brainstorming* with GF); 3rd Stage of the *DT -* Development (Profile Prototyping) and 4th Stage of the *DT -* Evaluation and Delivery (evaluation of evidence of validity).

### Stage 1 of the *DT* - Building Knowledge

It refers to the stage of research and search for an answer to a given problem, for which IR and documentary study were used. As a result, 26 publications were identified, of which the highest percentage (*n*=11; 42.3%) discussed the use of alcohol and drugs by young people, followed by family relationships (*n*=9; 34.6%) and the lowest number articles was related to sleep and rest with only 11.5%. 

With regard to technologies as an intervention to promote the health of these young people, it was found that of the 26 publications, only nine described them, three showing satisfactory results from the use of multimedia campaigns, two from *Facebook®* and healthy living programs, respectively. Next, the documentary study showed that 77.3% (*n*=649) of Portuguese young people and 62.6% (*n*=77) of Brazilian young people reported moderate or high satisfaction with their lives; 42.1% (*n*=405) said they often felt satisfied with the way they were and 30.5% (*n*=293) said they moderately felt ; 31.9% (*n*=307) said they sometimes worried about their bodies and 30.5% (*n*=293) said that they often felt worried about this; 55.4% (*n*=465) of Portuguese young people and 43.1% (*n*=53) of Brazilian young people expressed the desire to change something about their bodies as sometimes and often, respectively.

### Stage 2 of the *DT -* Product Definition (IR and *Brainstorming* with GF)

During the second stage, with regard to the second integrative review of the research, a sample 50 studies was obtained, the outcomes of which were analyzed, the most frequently addressed themes captured, arranged and categorized in the form of a word cloud. The most prominent themes were weight, perception, women, overweight and obesity, behavior, self-esteem and psycho-emotional aspects such as anxiety and depression. These results are essential for defining the product, as they highlight the areas that need to be addressed as a priority. Therefore, the product should include techniques that promote the psycho-emotional factors described and, at the same time, improve the perception of self-esteem, body image and the control of eating habits. Thus, through *brainstorming* with the GF, the aim is to explore innovative solutions that respond to these needs, in order to promote a positive impact on mental health, as well as on the well-being of young university students.

### Stage 3 of *DT* - Development (Profile Prototyping)

The GF, the final product was the creation of a profile on the social network *Instagram®* as a strategy for health promotion and education, which communicates from young person to young person through the creation of an *avatar*, where topics related to the self-perception and self-image of young people/adults were discussed. For this prototyping development, meetings were held with the development team responsible for building the *avatar*, where the need to generate more empathy with the young audience and representativeness in relation to insecurities linked to self- image was raised.

**Figure 1:**
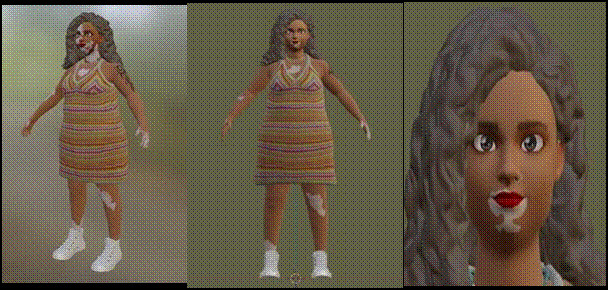
First built version of the avatar. Fortaleza, Ceará, Brazil, 2022

Thus, as a result of the discussions, it was suggested that the avatar had vitiligo (a pathology that causes depigmentation of the skin in the form of patches), making it clear from his image that he would face challenges related to self-image: *Why not include a chronic condition in this student?! P6; Yes... great idea, it could be a skin condition, for example vitiligo... what do you think?’ P8.* The other participants agreed and one student highlighted overweight as one of the most frequent complaints: ‘I think that overweight can also contribute, as it's one of the main complaints in our environment. P2. To do this, the researchers created a base model using the *ReadyPlayer®* tool, but due to the limited functionalities offered by the tool, it wasn't possible to include some of the necessary physical characteristics, such as being overweight, tattoos*, piercings* and lesions resulting from vitiligo, which were then edited by the development team using Zbrush® and Blender® *software* (Figure 1).

As a result, an *Instagram®* account was created with the name *@multi.brasilportugal.* The name was chosen to reflect the name of the technology proposed by the Multicentric Project (multi.) and the two countries participating in the research.

**Figure 2:**
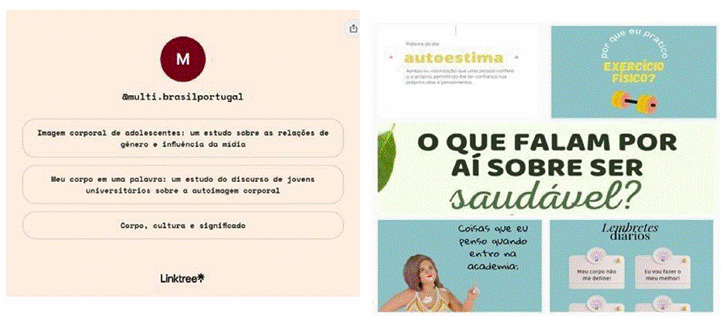
Social media profile and Linktr.ee. Fortaleza, Ceará, Brazil, 2022

Note: Image on the left with links for theoretical deepening of the theme. Image on the right with posts of scientific content related to self-care concerning self-image and body self-perception

On the profile page, there are five posts (four publications and one *Reel*) with themes related to health and self-image, as well as a *link to* the *Linktree®* tool (https://linktr.ee/multi.brasilportugal). This tool is a platform created to be a facilitating option for *Instagram®* users*,* grouping several *links* into just one (Figure 2). The *@multi.brasilportugal* profile provides *links* to the studies used as a basis for the content of the posts described in the next topic.

To define the themes to be covered in the posts, the results obtained in the third stage were used as a basis. Thus, the following themes were defined: distorted perception of weight and its prevalence in women; overweight, obesity and self-esteem; dissatisfaction with self-image and physical exercise; dissatisfaction with self-image and unhealthy eating behaviors. Each theme had a post on the *Instagram®* profile *feed*, in a carousel format, as well as *stories* and *reels* for some of them. In order to facilitate communication with young people, informal language and *emojis* were used. The images were produced by the researchers with the support of the *Canva®* tool, which offers design resources to help users produce content.

### Stage 4 of the *DT -* Evaluation and Delivery (evaluation of evidence of validity)

As for the assessment of the perceived usefulness of the technology (Table 1), four evaluators considered it slightly likely and two considered it extremely likely that the technology would allow them to carry out health promotion activities more quickly; three considered it slightly likely that it would improve their performance in health promotion actions; two considered it fairly likely that the technology would increase productivity during health promotion activities and the same proportion perceived it as slightly likely; two considered it fairly likely and the same proportion considered it slightly likely that its use would make it easier to carry out health promotion activities. Finally, three considered it extremely likely be useful in achieving its objective of promoting the health of young people/adults. 

**Table 1:** Assessment of the perceived usefulness of M-Health technology. Brazil, Portugal, 2022

Questions	Answers	***n*=7**
1. Using *@multi.brasilportugal* with young university students would allow me to carry out health promotion tasks more quickly.	Extremely likely	2
	Quite likely	1
	Slightly likely	4
2. Using *@multi.brasilportugal* would improve my performance in promoting the health of young university students.	Extremely likely	1
	Quite likely	1
	Slightly likely	3
3. Using *@multi.brasilportugal* with young university students would increase my productivity during health promotion activities.	Extremely likely	2
	Slightly likely	3
	Probably not	1
	Extremely unlikely	1
4. Using *@multi.brasilportugal* would increase my effectiveness in promoting the health of young university students.	Extremely likely	1
	Quite likely	2
	Slightly likely	2
	Probably not	1
5. Using *@multi.brasilportugal* would facilitate my health promotion activities with young university students.	Extremely likely	1
	Quite likely	2
	Slightly likely	2
	Probably not	1
	Extremely unlikely	1
6. I would find *@multi.brasilportugal* useful for promoting the health of young university students.	Extremely likely	3
	Quite likely	1
	Slightly likely	1
	Quite unlikely	1
	Extremely unlikely	1

Regarding the perception of ease of use (Table 2), three found it quite likely and two extremely likely to learn how to easily operate the *Instagram®* profile, with three already finding the technology easy to use. As for clarity and understanding of interaction with the technology, three considered it quite likely. And finally, in the same proportion of two, the evaluators considered it extremely likely and quite likely that it would be easily become adept at using it. As for the of achieving the purpose proposed by the technology, three found it quite likely, while two found it quite unlikely. With regard to flexibility in interacting with the technology, two thought it was quite likely, as well as slightly likely. However, the same proportion considered it slightly unlikely.

**Table 2:** Assessment of the perceived ease of use of the M-Health technology produced. Brazil, Portugal, 2022

Questions	Answers	***n*=7**
1. Learning how to operate *@multi.brasilportugal* would be easy for me.	Extremely likely	2
	Quite likely	3
	Slightly likely	1
	Extremely unlikely	1
2. I would find it easy to get *@multi.brasilportugal* to do what it sets out to do (promote the health of young university students).	Extremely likely	1
	Quite likely	3
	Slightly likely	1
	Quite unlikely	2
	Extremely likely	1
3. My interaction with *@multi.brasilportugal* would be clear and understandable.	Quite likely	3
	Slightly likely	1
	Probably not	1
	Slightly unlikely	1
4. I would find *@multi.brasilportugal* flexible to interact with.	Extremely likely	1
	Quite likely	2
	Slightly likely	2
	Slightly unlikely	2
5. It would be easy for me to become proficient in using *@multi.brasilportugal.*	Extremely likely	2
	Quite likely	2
	Slightly likely	1
	Slightly unlikely	1
	Extremely unlikely	1
6. I would find *@multi.brasilportugal* easy to use.	Extremely likely	2
	Quite likely	3
	Probably not	1
	Extremely unlikely	1

**Figure 3:**
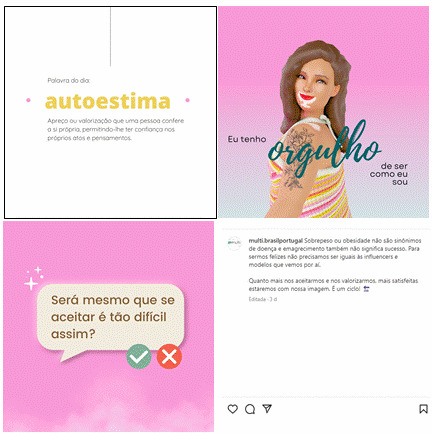
Publications and captions. Brazil and Portugal, 2022

Note: Images that represent posts of encouragement and interaction with young people. The aim is to contribute to self-pride and the self-acceptance process in order to improve self-esteem, self-image, and body self-perception.

With regard to the posts (Figure 3), the focus was to bring up the discussion about current beliefs, often disseminated by society and brought up as evidence in the studies identified, and thus enable their gradual deconstruction and the development of a new outlook for young people.

The second image, on the other hand, aims to promote the *"Make peace with the mirror"* movement, encouraging self-acceptance regardless of one's physical characteristics, breaking with the aesthetic standards imposed by society. The subtitles explain a little more about the topics and also give young people the chance to consult the bibliographical references used to prepare the content and deepen their knowledge. Thus, one of the dynamic images produced was used, which shows the persona looking at her body, with its characteristics that can socially be considered non- standard. The caption briefly describes the results of this study, which can be accessed via the *link* provided on the profile. 

A second strategy for discussion on the subject was to create a post in the form of a *reel*, which consists of a short video with the use of a song. The aim was to address beliefs described by young people related to the feeling of shame and not belonging in environments geared towards physical activity, such as gyms. *Reels* can be considered one of the most effective strategies for reaching a large number of users today, due to the configuration of the social network algorithm.

**Figure 4:**
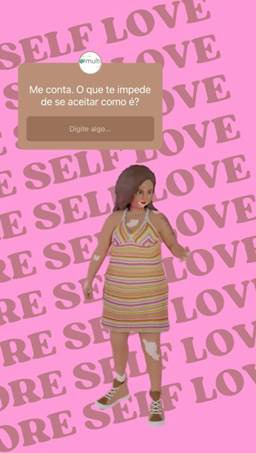
Dynamic story. Brazil and Portugal, 2022

Note: Image representing an interactive and dynamic story (Instagram story) seeking to understand the barriers to self-acceptance.

Polls were also created in the form of *stories* on the profile, the aim of which is to find out more about users' perceptions of a particular subject and thus use this information to create content that is geared to their real needs (Figure 4).

After evaluating the *Instagram* profile, it was possible to register it as a technical-technological product licensed under Creative Commons as: “*Instagram* profile @multi.brasilportugal © 2022 by Lia Araruna de Lima & Fernanda Jorge Magalhães is licensed under Creative Commons Attribution-NonCommercial-NoDerivatives 4.0 International.”

## Discussion

Following the recommendation of the FG participants, the *Instagram®* profile begins with a brief presentation of the persona behind the profile, with the aim of contextualizing the scenario, clarifying the purpose of the profile and beginning to create a bond with the young user. The decision to build her as a woman with vitiligo was based on the fact that it is a pathology still shrouded in stigma and prejudice, especially when it affects women, due to the possibility of causing dissatisfaction with self-image, intense psychological distress and serious problems such as depression and social isolation.^11^ As mentioned above, the content of the posts was defined on the basis of the results identified in the third stage. On *the theme "Distorted perception of weight and its prevalence in women",* a post was created in the format of a carousel, or that is, more than one image contained in the *post*, the first of which displays daily reminders, which were constructed from the speeches of young people collected in some of the publications found.^12^ Its aim is to promote the breaking down of concepts.

The next post, on the subject of *"Overweight, obesity and self-esteem"*, discusses the subject of self-esteem and its relationship with body shape and dissatisfaction with it, as described in some of the studies presented above.^13^ Three images associated with the theme were produced with a related caption arranged in a carousel format. The images begin with the concept of self-esteem, since it is closely linked to body satisfaction.^14^ Next, the persona promotes self-acceptance through its positioning and, finally, displays a question for the user to reflect on. In addition to this post, we used the *stories* format and the question box tool offered by *Instagram®* to promote communication with users. Young people tend to be more comfortable in the virtual space, feeling free to express their opinions, insecurities and desires anonymously or not.^15^

The fourth post reflects on the meaning of physical exercise, going beyond its use to obtain the perfect body. To do this, it used the strategy of listing reasons that can encourage young people to exercise, according to discourses observed in a study on the subject.^16^ The fifth and final post addresses the myths involved in the concept of healthy eating and healthy bodies discussed in publications. To do this, four static images and a caption were created to promote the movement of *"making peace"* with food (Figure 3). With this figure it is possible to discuss the concept of healthy eating, the divergence from the imposed obligation of thinness as a synonym for health and the encouragement of balance when eating.^13^^,^^17^ As well as the use of digital tools with current generations, in order to bring closer together and make them useful for reaching young people, especially due to the flexibility, convenience and portability of technology, combined with the familiarity of such platforms and social media.^18^

The lives of millions of people around the world have already changed, and will continue to change, as a result of the impact of mobile technologies.^19^ Young people can be called digital natives, and the ease of access and the amount of technological resources available today can act as a bridge to connect them with health systems.^20^ It is also essential to highlight how challenging it is to develop *mHealth* technology that is actually effective in changing behavior and requires more than easy handling and accessibility.^21^ The main purpose of an *mHealth* technology is to disseminate access to health information and services with a focus on promoting personal well-being, health prevention and chronic disease management.^22^ Or that is, it must be incorporated into a dynamic of prevention and health promotion.^23^

When evaluating the technology as useful for achieving its objective of promoting the health of young people/adults, it is known that, in order to change health- related risk behaviors, the health care model must be based on promoting discussions about the reasons that lead to the adoption of healthier behaviors.^24^ Therefore, putting on the agenda and discussing some beliefs rooted in today's society, and thus seeking to reframe them, is an important strategy for changing behaviors. When talking specifically about the *Instagram®* tool, it is perceived as an important, albeit relatively new, tool for health professionals to use in their business, and in the health education process, giving them more freedom to market their products, publicize specific items, in order to increase the likelihood of broadening and reaching a certain audience.^25^

A netnographic study carried out to investigate the work process of nurses on *Instagram* found that the tool also contributes to the dissemination of these professionals as freelancers, providing an innovative and effective approach to influence and being an excellent platform for disseminating important information, thus corroborating the results obtained.^26^ In this context, nurses play an important role in developing a reflexive awareness to protect and promote the health of individuals when, through activities that generate knowledge, they help to break down the concept of vertical transmission of information and generate a feeling of co-responsibility in making decisions about social and health behaviors.^27^

Conclusion. An *Instagram®* social network profile called *@multi.BrasilPortugal* was built and initially evaluated, with the aim of promoting the health of young Brazilians and Portuguese by strengthening their self-perception and self-image. The use of the *DT* was a very favorable strategy, since it enabled the ideation and prototyping processes to be carried out by young people and health experts of young people, getting to know their real pains and needs and making it possible to develop a technology that is more likely to achieve its results. It is suggested that the research be continued by other researchers, with the aim of carrying out evaluations with a larger sample, especially in Brazil, so that it can be made available to the general public and be a health promotion tool for both the target public and health professionals. Clinical validation studies of the technology are also suggested, with the target public and in the clinical practice of health education and health promotion.
